# Nutricional value of two protein hydolysates selected for the design of a new therapeutic infant formula

**DOI:** 10.1186/2045-7022-1-S1-P113

**Published:** 2011-08-12

**Authors:** Esther Matencio, Sergio Muñoz, Josune Olza, Saray Santamaría, Fernando Romero, Pedro Abellán, Angel Gil

**Affiliations:** 1 Hero Institute for Infant Nutrition, Research, Spain; 2 Institute of Nutrition and Food Technology. Centre of Biomedical Research University of Granada, Biochemistry and Molecular Biology II., Granada, Spain; 3 Hero Institute for Infant Nutrition, Research, Murcia, Spain

## Background

Breastfeeding is the gold standard of infant feeding, however not all infants with cow’s milk protein allergy (CMPA) tolerate human milk. In these cases, it seems recommended the use of extensively hydrolyzed formula (eHF). Preliminary analyses such as antigenicity and protein quality evaluation are needed to ensure that new formula will be nutritionally suitable, tolerated and safe for infants with CMPA. The aim of this study was to determine the protein quality of a whey hydrolysate (WH) and casein hydrolysate (CH).

## Methods

The Thomas-Mitchell method modified was used. Male Wistar rats weighing about 50 g were housed in metabolic cages and distributed in three groups fed with diets only differ in protein source. Group 1 fed with WH, group 2 fed with CH and group 3 fed with casein +5% DL-methionine reference diet. Acclimatization of five days followed of ten days with experimental diet. In the last seven days we controlled daily intakes and collected faeces and urine. For estimation of the protein quality, true digestibility, net protein utilization (NPU), biological value (BV) and protein efficiency ratio (PER), were used. Nitrogen content was measured by means of Kjeldahl method in diet, faeces and urine.

## Results

WH and CH showed similar NPU (67,18%, 69,55%), BV (72,99%, 75,26%) and PER (3,62, 3,69)). NPU, BV and PER in control diet were (81,01%, 85,64% and 4,14).Both hydrolysates complied with the requirements of PER higher than 2.5 and BV higher than 70, considered as adequate sources of amino nitrogen for human nutrition.

## Conclusions

WH and CH are good protein sources to be used in the design of a new eHF for the nutritional treatment of infants with CMPA.

